# Supplementary material for: Quantitative determination of perfluoroalkyl substances (PFAS) in soil, water, and home garden produce
Source: MethodsX. 2018 Jun 28;5:697–704. doi: 10.1016/j.mex.2018.06.017 (PMC6039355; doi:10.1016/j.mex.2018.06.017)
Supplement: Supplementary file 1 [file mmc1.docx]

Quantitative Determination of Perfluoroalkyl Substances (PFAS) in Soil, Water, and Home Garden Produce

*Carin A. Huset*, Kitrina M. Barry,* Virginia L Yingling*,* Richard W Hoffbeck*,* Rita B Messing*,* James E Kelly*,*

Minnesota Department of Health, Saint Paul, Minnesota

*Contact email: [carin.huset@state.mn.us](mailto:carin.huset@state.mn.us), [kitrina.barry@state.mn.us](mailto:kitrina.barry@state.mn.us), [virginia.yingling@state.mn.us](mailto:virginia.yingling@state.mn.us), [rhoffbeck@gmail.com](mailto:rhoffbeck@gmail.com), [messir1@live.com](mailto:messir1@live.com), [james.kelly@state.mn.us](mailto:james.kelly@state.mn.us),

Abstract:
This data article includes details on the simple and efficient analytical methods used to measure perfluoroalkyl substances (PFASs) in water, soil, and produce from home gardens in Minnesota. PFASs in water were analyzed via direct injection. PFASs were extracted from homogenized soil using sonication, and from produce using dispersive solid phase extraction. Isotope dilution was used for quantitation in all methods. The method performance parameters and quality control measures are described. The methods described are applicable for a PFAS ranging from C4-C8 and the produce method was used on a wide variety of produce. For further details and experimental findings, please refer to the article “Occurrence of perfluoroalkyl substances (PFAS) in garden produce at homes with a history of PFAS-contaminated drinking water” The key benefits of this method are:

This method adapts dispersive solid phase extraction for the analysis of PFAS in produce.

The method can be used to analyze PFAS ranging from 4 to 8 carbons in a variety of produce types.

**Keywords:**

Perfluorooctanoate, PFOA, perfluorooctane sulfonate, PFOS, perfluorobutanoate, PFBA, perfluoropentanoate, PFPeA, produce, dispersive solid-phase extraction, liquid chromatography, tandem mass spectrometry

**Specifications Table**

| Subject area | - *Environmental Science* |
| --- | --- |
| More specific subject area | *Quantitative analysis of PFAS in environmental matrices* |
| Method name | *Quantification of PFAS in produce using LC-ESI-MS/MS* |
| Name and reference of original method |  |
| Resource availability |  |

**Experimental Design, Materials and Methods**

**Sample Collection**

Collection of water, soil, and produce samples is described elsewhere. Water samples were refrigerated after collection and analyzed within 14 days. Produce and soil samples were frozen after collection until sample preparation and analysis. All unknown samples were at room temperature at the onset of sample preparation activities.

**Chemicals**

High purity chemical standards of perfluorobutanoic acid (PFBA), perfluoropentanoic acid (PFPeA), perfluorohexanoic acid (PFHxA), perfluorooctanoic acid (PFOA), perfluorobutane sulfonate (PFBS), perfluorohexane sulfonate (PFHxS), and perfluorooctane sulfonate (PFOS) were purchased from Wellington Laboratories (Wellington Laboratories, Guelph, Ontario, Canada). Internal standards, perfluoro-n-[^13^C_4_]butanoic acid (MPFBA), perfluoro-n-[^13^C_5_]pentanoic acid (MPFPeA), perfluoro-n-[1,2-^13^C_2_]hexanoic acid (MPFHxA), perfluoro-n-[1,2,3,4-^13^C_4_]octanoic acid (MPFOA), perfluoro-1- hexane[^18^O_2_]sulfonate (MPFHxS), and perfluoro-1-[1,2,3,4-^13^C_4_]octane sulfonate (MPFOS) were also purchased from Wellington Laboratories. Standards were >98% pure and isotopic purity was >99% for ^13^C and >94% for ^18^O. Acetonitrile, formic acid, methanol and ammonium hydroxide were purchased from Fisher (Fisher Scientific, Pittsburgh, PA). Supelclean Envicarb 120/400 was purchased from Supelco (Sigma-Aldrich, Bellefonte, PA). Magnesium Sulfate Heptahydrate was purchased from VWR and muffled in house (VWR, Radnor, PA).

**Sample preparation**

Aliquots of water samples (150 µL) were transferred to 2 mL polypropylene autosampler vials and spiked with internal standards in ACN (50 µL of 3 ng/mL stock) and then analyzed.

Soil samples were air dried and sieved with a #16 stainless steel sieve (a portion was retained for wet weight determination). Soil was extracted based on previously reported methods(Sepulvado, Blaine et al. 2011). Briefly, aliquots of soil (5 g each) were spiked with internal standard (50 µL of 100 ng/mL stock) and then extracted by sonication and shaking with three rounds of 7 ml methanol with 1% ammonium hydroxide. Supernatants were removed, combined and concentrated to dryness under nitrogen. After reconstitution with two rounds of 0.5 mL acetonitrile, extracts were cleaned up using graphitized carbon black (25-40 mg) and 100 µL transferred to an autosampler vial and diluted with 25 µL ACN and 375 µL reagent water for analysis.

The edible parts of the produce (skins, stems, seeds removed, as appropriate) were homogenized and 5 g aliquots were extracted with a dispersive solid phase extraction, or QuEChERS, method(Anastassiades and Lehotay 2003). Internal standards (50 µL of 25 ng/mL stock) were added to samples and 2 g of magnesium sulfate and three rounds of 10 mL acetonitrile with 1% ammonium hydroxide were used in the extraction. The supernatants were combined and concentrated to dryness with nitrogen. Extracts were then reconstituted with two rounds of 350 µL acetonitrile and cleaned up with graphitized carbon black (25-40 mg) prior to analysis. For analysis, 70 µL of the extract was transferred to an autosampler vial and diluted with 55 µL ACN and 375 µL reagent water.

**Analysis**

All prepared samples or extracts were analyzed using an Agilent 1100 HPLC (Santa Clara, California) and a Waters Quattro Micro tandem mass spectrometer (Milford, Massachusetts). Aliquots of prepared samples or extracts were injected onto the HPLC system and separated via reversed phase chromatography. HPLC conditions and gradients used are found in Tables 1 – 3. The tandem mass spectrometer was operated in ESI- mode, with multiple reaction monitoring windows used to maximize sensitivity. Tandem mass spectrometer (MS/MS) conditions are found in Tables 4 and 5. Multiple transitions, when available, were monitored for each analyte and multiple reaction monitoring (MRM) was used to maximize signal for each analyte. (Table 6).

Table 1.

General HPLC conditions

| Analytical Column | Thermo Betasil C8, 50 x 2.1 mm, 3 μm  with Upchurch PEEK 0.5 μm prefilter |
| --- | --- |
| Guard Column | Thermo Betasil C8 3.0 x 30 mm, 5 µm |
| Column Temperature | 30 ^o^C |
| Sample Temperature | 5 ^o^C |
| Injection Volume | 10-20 μL |
| Mobile Phase A | 0.1% formic acid in water |
| Mobile Phase B | 0.1% formic acid in acetonitrile |

Table 2.

HPLC conditions for PFPeA, PFHxA, PFOA, PFBS, PFHxS, and PFOS

| Time  (min) | % mobile  Phase A | % mobile  Phase B | Flow rate  (μL/min) |
| --- | --- | --- | --- |
| 0 | 65 | 35 | 0.4 |
| 0.25 | 50 | 50 | 0.4 |
| 3.0 | 10 | 90 | 0.4 |
| 4.0 | 10 | 90 | 0.7 |
| 5.75 | 10 | 90 | 0.7 |
| 5.76 | 65 | 35 | 0.7 |
| 6.25 | 65 | 35 | 0.7 |
| 6.5 | 65 | 35 | 0.4 |
| 7 | 65 | 35 | 0.4 |

Table 3.

HPLC conditions for PFBA

| Time  (min) | % mobile  Phase A | % mobile  Phase B | Flow rate  (μL/min) |
| --- | --- | --- | --- |
| 0 | 70 | 30 | 0.4 |
| 2.0 | 70 | 30 | 0.4 |

Table 4.

MS/MS source conditions

| Source | Set |
| --- | --- |
| Polarity | ES- |
| Capillary (kV) | 0.40 |
| Extractor (V) | 1 |
| RF Lens (V) | 0.2 |
| Source Temperature (^o^C) | 120 |
| Desolvation Temperature (^o^C) | 350 |
| Desolvation Gas Flow (L/hr) | 700 |
| Cone Gas Flow (L/hr) | 0 |

Table 5.

MS/MS analyzer parameters

| Analyzer | Set |
| --- | --- |
| LM1 Resolution | 10.0 |
| HM1 Resolution | 10.0 |
| Ion Energy 1 | 1.0 |
| Entrance | -5 |
| Collision | 15 |
| Exit | 1 |
| LM2 Resolution | 13.0 |
| HM2 Resolution | 13.0 |
| Ion Energy 2 | 1.5 |
| Multiplier (V) | 750 |
| Gas Cell Pirani Pressure (mbar) | 3.0 e-3 |

**Table 6.**

MS/MS acquisition parameters

| MRM transitions | | | | | | |
| --- | --- | --- | --- | --- | --- | --- |
| Analytes | Q1 > Q3 | RT (min) | Dwell (s) | Cone (V) | CE (eV) | Delay (s) |
| PFBA^a^ | 212.9 > 168.9 | 1.3 | 0.20 | 18.0 | 9.0 | 0.01 |
| PFPeA^a^ | 262.8 > 219.1 | 1.6 | 0.20 | 16.0 | 9.0 | 0.01 |
| PFHxA1^a^ | 312.7 > 269.0 | 2.5 | 0.10 | 15.0 | 9.0 | 0.01 |
| PFHxA2 | 312.7 > 118.9 | 2.5 | 0.05 | 15.0 | 21.0 | 0.01 |
| PFOA1^a^ | 412.6 > 369.0 | 3.7 | 0.10 | 18.0 | 10.0 | 0.01 |
| PFOA2 | 412.6 > 169.2 | 3.7 | 0.05 | 18.0 | 18.0 | 0.01 |
| PFBS1^a^ | 298.7 > 79.8 | 2.9 | 0.10 | 45.0 | 29.0 | 0.01 |
| PFBS2 | 298.7 > 98.8 | 2.9 | 0.05 | 45.0 | 29.0 | 0.01 |
| PFHxS1^a^ | 398.6 > 79.8 | 4.0 | 0.10 | 50.0 | 35.0 | 0.01 |
| PFHxS2 | 398.6 > 98.8 | 4.0 | 0.05 | 50.0 | 30.0 | 0.01 |
| PFOS1^a^ | 498.5 > 79.8 | 4.9 | 0.10 | 60.0 | 45.0 | 0.01 |
| PFOS2 | 498.5 > 98.9 | 4.9 | 0.10 | 60.0 | 40.0 | 0.01 |
| MPFBA^b^ | 216.9 > 172.1 | 1.3 | 0.10 | 15.0 | 10.0 | 0.01 |
| MPFPeA^b^ | 267.8 > 223.1 | 1.6 | 0.20 | 15.0 | 9.0 | 0.01 |
| MPFHxA^b^ | 314.6 > 270.0 | 2.5 | 0.10 | 15.0 | 10.0 | 0.01 |
| MPFOA^b^ | 416.7 > 371.9 | 3.7 | 0.10 | 15.0 | 11.0 | 0.01 |
| MPFHxS^b^ | 402.6 > 83.8 | 4.0 | 0.10 | 55.0 | 35.0 | 0.01 |
| MPFOS^b^ | 502.5 > 79.9 | 4.9 | 0.10 | 60.0 | 40.0 | 0.01 |

^a^ Primary transition used for quantitation. Secondary transitions (when available) used for confirmation
^b^ Stable isotope label internal standards

**Produce Categories**

Due to the wide variety of produce grown by study participants, produce was categorized into four types for the purposes of analysis (Table 7). These types are high acidity group, high water content group (>90% water content), low water content group (<90% water content), and leafy herbs and greens. Classification of high acidity or leafy herbs and greens was more important than classification by water content; therefore tomatoes are in the high acidity group and lettuce is in the leafy herbs and greens group, and not the high water content. Quality control and performance studies were performed on a representative type of produce from each group.

**Table 7.**

Produce groups with percent water^a^

| Group 1: High Acidity | Group 2: High water content | Group 3: Low water content | Group 4: Leafy herbs & greens |
| --- | --- | --- | --- |
| Apple (86%) | Asparagus (93%) | Acorn squash (88%) | Basil (92%) |
| Blackberry (88%) | Beans (90%) | Beet (88%) | Cabbage (92%) |
| Grape (81%) | Cantaloupe (90%) | Broccoli (89%) | Celery (95%) |
| Raspberry (86%) | Cauliflower (92%) | Brussel sprout (86%) | Chives (91%) |
| Rhubarb (94%) | Cucumber (95%) | Butternut squash (86%) | Dill (86%) |
| Strawberry (91%) | Eggplant (92%) | Carrot (88%) | Fennel (*) |
| **Tomato (95%)** | Kohlrabi (91%) | Horseradish (88%) | **Lettuce (95%)** |
|  | **Bell pepper (94%)** | Kale (84%) | Mint (80%) |
|  | Radish (95%) | Leek (83%) | Oregano (*) |
|  | Summer squash (95%) | Onion (89%) | Parsley (88%) |
|  | Watermelon (91%) | **Peas (89%)** | Rosemary (68%) |
|  | Zucchini (95%) | Hot & sweet peppers (88%) | Spinach (91%) |
|  |  | Potato (79%) | Swiss chard (93%) |
|  |  | Sweet corn (76%) | Thyme (65%) |
|  |  | Shallot (80%) |  |

^a^ Percent water is calculated based on values from the USDA (<http://www.nal.usda.gov/fnic/foodcomp/search/>). Acidity is based on <http://www.engineeringtoolbox.com/food-ph-d_403.html> Produce types in bold were determined to be representative of their group and were used for method development, method performance, and quality control purposes.

*Fennel and Oregano water content unavailable, placed in Leafy herbs and greens group based on similar characteristics

**Quantitation and Quality Control**

Calibration curves were prepared daily from stock solutions and quantitation was achieved through isotope dilution. Calibration ranges are listed in Table 8, and curves were constructed with 1/x weighting for all analytes.

**Table 8.**

Calibration ranges^a^

|  |  |  | Produce | | | |
| --- | --- | --- | --- | --- | --- | --- |
|  | Water (µg/mL) | Soil (µg/kg) | High acidity  (µg/kg) | High water content (µg/kg) | Low water content (µg/kg) | Leafy greens & herbs (µg/kg) |
| PFBA | 0.05-10 | 0.75-50 | 0.1-40 | 0.05-40 | 0.05-40 | 0.05-40 |
| PFPeA | 0.05-10 | 0.75-10 | 0.05-3 | 0.05-3 | 0.05-3 | 0.1-3 |
| PFHxA | 0.05-10 | 0.10-10 | 0.1-3 | 0.05-3 | 0.05-3 | 0.1-3 |
| PFOA | 0.05-10 | 0.10-10 | 0.1-3 | 0.05-3 | 0.05-3 | 0.05-3 |
| PFBS | 0.05-10 | 0.10-10 | 0.05-3 | 0.05-3 | 0.05-3 | 0.05-3 |
| PFHxS | 0.05-10 | 0.75-10 | 0.05-3 | 0.05-3 | 0.05-3 | 0.05-3 |
| PFOS | 0.05-10 | 0.75-50 | 0.05-3 | 0.05-3 | 0.05-3 | 0.05-3 |

^a^ For water samples exceeding the highest level, a new aliquot is diluted to be in the calibration range and reanalyzed.

Ongoing quality control samples were prepared, extracted, and analyzed with each extraction batch (up to 20 unknown samples). All quality control samples except the calibration verification and report limit verification standards were treated like unknown samples. Method specific limits can be found in Table 9.

### Table 9.

### Ongoing quality control composition, limits, and frequency

|  | Composition | | Percent Recovery Limits | | | Frequency per batch | | RPD^b^ limits |
| --- | --- | --- | --- | --- | --- | --- | --- | --- |
| Matrix | MB | LCS | CVS | LCS | MS | MSD | Dup | Dup or MSD |
| Water | reagent water | spiked reagent water | 80-120% | 80-120% | 70-130% | 1 of either | | ≤ 20% |
| Soil | wet sand | spiked wet sand | 70-130% | 50-150% | 50-150% | 1 | 1 | ≤ 50% |
| Produce | reagent water | spiked produce | 70-130% | 60-130% | 50-150% | 1^a^ | 1^a^ | ≤ 50% |

^a^ Per produce type if possible

^b^ RPD: Relative Percent Difference

A calibration verification standard (CVS) was analyzed for every 24-hour analysis period. The CVS is a mid-level standard that is not extracted. When possible, the spiking solution for the CVS should be prepared from a different vendor lot than the calibration standards. The internal standard solutions may be from the same lot. At a minimum it was analyzed at the end of an analytical sequence and every twelve hours during analytical runs.

A method blank (MB) was analyzed with every batch of samples. It was treated like an unknown sample and is used to demonstrate that there are no interferences or contamination being introduced by the steps of the method that might result in false positives. The composition varied by method and is listed in Table 9.

A laboratory control sample (LCS) was analyzed with every batch of unknown samples. The purpose of the LCS was to verify that the procedure was in control and that the laboratory is capable of making accurate measurements. The composition and control limits of the LCS varied by method and can be found in Table 9. For all matrices, the sample was spiked with a known level of analytes and the percent recovery calculated. The relative percent difference (RPD) between the measured value and the value determined during method validation was calculated for all analytes within the calibration range and used in place of percent recovery.

Matrix spikes (MS), aliquots of unknown samples that were spiked with a known amount of the analytes, were run in each method. For water and soil a MS was analyzed for every unknown sample (sample amounts permitting). The produce method analyzed one MS per type of produce in a batch. The calculated concentration of the spiked sample was compared to the theoretical value. The limits varied by method and can be found in Table 9. Failure to meet this criterion indicated significant matrix interference, and when possible that particular sample was diluted and reanalyzed. It should be noted that since this method is a dilution method, the spiked analytes are primarily testing for suppression and enhancement of the target ions within the triple quadrupole mass spectrometer.

Sample duplicates (DUP) and matrix spike duplicates (MSD) were analyzed and the percent relative percent deviation calculated. Frequency and acceptance limits varied by method and can be found in Table 9.

For the water method only, a report level verification standard was analyzed to determine that the report level was valid for a target analyte and a given analytical run. For an acceptable analysis, the percent recovery for all analytes was within 70 to 130%.

The produce method also included a matrix blank (MXBL), consisting of representative produce, that was analyzed with every batch for every produce group in the batch. Due to the difficulty in finding blank produce, results for the MXBL were not always blank. All analyte concentrations were within 50% to 200% of the concentration initially determined for that sample of representative produce (often determined during Method Detection Limit (MDL) or validation studies and based on at least 5 replicate measurements).

**Method Performance**

Method detection limits (MDL) were established during validation and were determined from measurement of a minimum of seven replicates at a concentration estimated to be two to five times the noise level. Representative produce types for the four groups were used as described above. All replicates are processed through each step contained in the method. MDL is calculated using the equation below, where SD is the standard deviation of the replicates, t is the student’s t value at 99% confidence interval and n is the number of replicates. The MDL for each matrix can be found in Table 10.

*MDL* = *SD*(*t*_(n-1)_)

Accuracy and precision were determined through spike and recovery experiments. Aliquots of each matrix were spiked to approximately mid-range on the calibration curve and extracted and analyzed as described above. Since we were unable to obtain blank produce and soil for the spike and recovery experiments, additional aliquots of each matrix were also spiked with internal standards only extracted and analyzed as described above. The accuracy, represented by percent recovery, and precision, represented by the relative standard deviation, for each analyte in each matrix is listed in Table 10.

**Table 10.**

Method performance ^a^

| Analyte | Tomato  (high acidity) | | Lettuce  (leafy greens & herbs) | | Bell Pepper  (high water content) | | Peas  (low water content) | | Soil | | Water | |
| --- | --- | --- | --- | --- | --- | --- | --- | --- | --- | --- | --- | --- |
|  | Accuracy (precision) | MDL (µg/kg) | Accuracy (precision) | MDL (µg/kg) | Accuracy (precision) | MDL (µg/kg) | Accuracy (precision) | MDL (µg/kg) | Accuracy (precision) | MDL (µg/kg) | Accuracy (precision) | MDL (µg/L) |
| PFBA | 96 (4.7) | 0.018 | 94 (3.6) | 0.015 | 87 (6.5) | 0.013 | 97 (1.8) | 0.015 | 99 (1.33) | 0.008 | 111 (1.7) | 0.004 |
| PFPeA | 87 (6) | 0.011 | 84 (6.3) | 0.008 | 90 (3.4) | 0.008 | 84 (1.8) | 0.021 | 101 (1.47) | 0.016 | 93 (1.8) | 0.003 |
| PFHxA | 100 (8) | 0.021 | 90 (3.4) | 0.011 | 96 (6.3) | 0.016 | 97 (5.8) | 0.018 | 102 (2.35) | 0.018 | 103 (2.4) | 0.004 |
| PFOA | 92 (10) | 0.029 | 94 (7.7) | 0.011 | 100 (7) | 0.010 | 94 (5.5) | 0.013 | 101 (1.75) | 0.033 | 108 (2.9) | 0.004 |
| PFBS^b^ | 63 (5.3) | 0.012 | 56 (8.6) | 0.006 | 80 (4.3) | 0.009 | 70 (2.1) | 0.008 | 116 (7.06) | 0.024 | 96 (2.3) | 0.006 |
| PFHxS | 98 (2.6) | 0.013 | 91 (2.8) | 0.010 | 95 (1.7) | 0.003 | 99 (6.7) | 0.008 | 99 (2.26) | 0.011 | 95 (1.3) | 0.003 |
| PFOS | 79 (5.5) | 0.008 | 78 (4) | 0.008 | 84 (9) | 0.007 | 80 (3.9) | 0.011 | 103 (0.84) | 0.012 | 101 (2.5) | 0.004 |

^a^ MDL soil: 0.075 µg/kg spike , n=9 spiked, n=5 unspiked; MDL produce: 0.05 µg/kg spike, n=9 spike for tomato (n=5 for PFBA), n=8 for lettuce, bell pepper, peas; MDL water: 0.025 µg/L spike, n=7; Water spike: 2.5 µg/L (n=7); Soil spike: 1 µg/kg (n=6 spiked, n=5 unspiked); Produce spike: 0.25 µg/kg (n=5 spiked, n=4 unspiked for tomato; n=5 spiked, n=3 unspiked for lettuce, bell pepper, peas)

^b^ No stable isotope labeled internal standard was available for PFBS. Wider acceptance limits were used for spike and recovery experiments.

Acknowledgements

This study was funded by the Minnesota Pollution Control Agency and the Minnesota Department of Health. The authors gratefully acknowledge the study volunteers for allowing MDH staff to visit their homes several times to collect samples and administer surveys.

References:

Anastassiades, M. and S. J. Lehotay (2003). "Fast and Easy Multiresidue Method Employing Acetonitrile Extraction/Partitioning and "Dispersive Solid-Phase Extraction" for the Determination of Pesticide Residues in Produce." Journal of AOAC International **86**(2): 412-431.

Sepulvado, J. G., et al. (2011). "Occurrence and Fate of Perfluorochemicals in Soil Following the Land Application of Municpal Biosolids." Environmental Science and Technology **45**(19): 8106-8112.
